# A key driver to promote HCC: Cellular crosstalk in tumor microenvironment

**DOI:** 10.3389/fonc.2023.1135122

**Published:** 2023-03-15

**Authors:** Pengyue Liu, Lingyu Kong, Ying Liu, Gang Li, Jianjia Xie, Xin Lu

**Affiliations:** ^1^ Clinical Medical College, North China University of Science and Technology, Tangshan, China; ^2^ Department of Traditional Chinese Medicine, Affiliated Hospital of North China University of Science and Technology, Tangshan, China; ^3^ Department of Clinical Skills Training Center, Tangshan Gongren Hospital, Tangshan, China; ^4^ Department of Clinical Laboratory, Tangshan Maternal and Child Health Care Hospital, Tangshan, China

**Keywords:** hepatocellular carcinoma (HCC), tumor microenvironment (TME), tumor malignant progression, cellular crosstalk, tumor immunosuppression

## Abstract

Liver cancer is the third greatest cause of cancer-related mortality, which of the major pathological type is hepatocellular carcinoma (HCC) accounting for more than 90%. HCC is characterized by high mortality and is predisposed to metastasis and relapse, leading to a low five-year survival rate and poor clinical prognosis. Numerous crosstalk among tumor parenchymal cells, anti-tumor cells, stroma cells, and immunosuppressive cells contributes to the immunosuppressive tumor microenvironment (TME), in which the function and frequency of anti-tumor cells are reduced with that of associated pro-tumor cells increasing, accordingly resulting in tumor malignant progression. Indeed, sorting out and understanding the signaling pathways and molecular mechanisms of cellular crosstalk in TME is crucial to discover more key targets and specific biomarkers, so that develop more efficient methods for early diagnosis and individualized treatment of liver cancer. This piece of writing offers insight into the recent advances in HCC-TME and reviews various mechanisms that promote HCC malignant progression from the perspective of mutual crosstalk among different types of cells in TME, aiming to assist in identifying the possible research directions and methods in the future for discovering new targets that could prevent HCC malignant progression.

## Introduction

Liver cancer is the third greatest cause of cancer-related mortality (1). The most common pathological type of primary liver cancer is HCC, which accounts for more than 90% (2). HCC is characterized by high morbidity and mortality and has been a heavy burden for the public health system worldwide (3). Moreover, due to the fact that HCC is predisposed to metastasize, reappear and occur resistant to treatment, the benefits from conventional therapies such as surgical resection, radiofrequency ablation as well as transarterial chemoembolization are limited (4). It is involved in multiple mechanisms ranging from gene mutation and epigenetic alterations to complex cellular crosstalk and signaling pathways which cause abnormal accumulation and function of certain molecules and cells in tumor tissue and ultimately result in HCC malignant progression. Several clinical trials have verified that many kinds of tyrosine kinase inhibitors (TKIs) such as sorafenib and cabozantinib provide survival benefits in HCC patients (5–7) and that several immune checkpoint inhibitors (ICIs) such as nivolumab and pembrolizumab have potential for advanced HCC therapy (8, 9). Moreover, the immunotherapy combining ICIs with other treatments such as kinase inhibitors, anti-angiogenic drugs shows great prospects in the treatment of HCC (10). Nevertheless, the proportion of HCC patients responding to them is very low because of the high genetic, epigenetic heterogeneities and the formation of immunosuppressive TME (11, 12). Hence, HCC is still a highly fatal tumor as it is very predisposed to frequent recurrence and distant metastasis after surgery (13).

**Figure 1:**
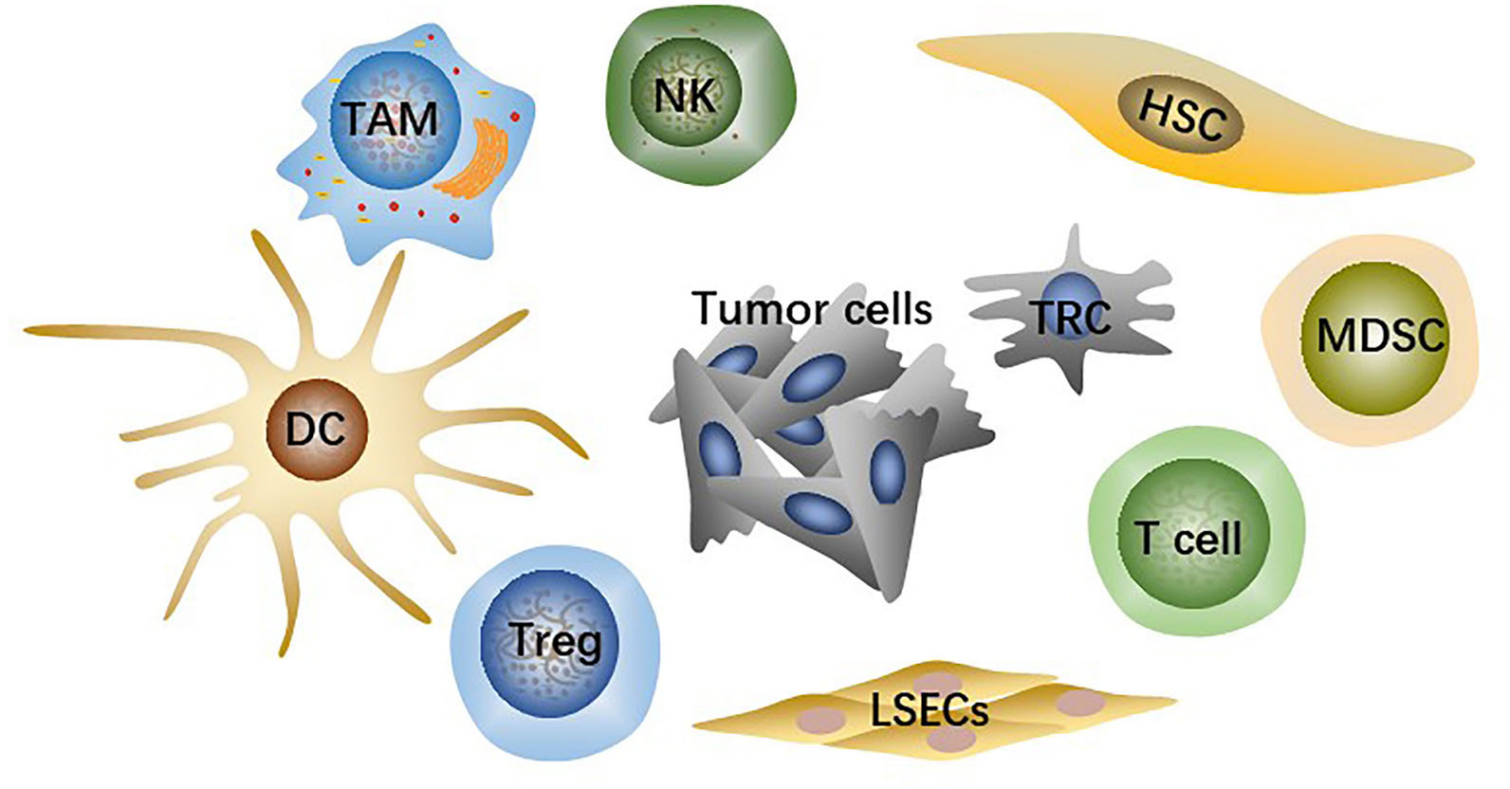
The main cell types in TME include tumor cells, effector T cells, NK cells, dendritic cells (DCs), hepatic stellate cells (HSCs), liver sinusoidal endothelial cells (LSECs), tumor-associated macrophages (TAMs), regulatory T cells (Tregs), myeloid-derived suppressor cells (MDSCs).

TME is the microsystem that supports tumor cells survival and tumor progression and is always regulated by cellular metabolism, genetic and epigenetic factors. Besides tumor cells, the HCC-TME consists of adaptive and innate immune cells, stromal cells, and liver sinusoidal endothelial cells (LSECs) (**Figure 1**) as well as non-cellular components such as cytokines and signaling proteins secreted by the cells above. Immunosuppressive innate immune cells include tumor-associated macrophages (TAMs), regulatory T cells (Tregs), myeloid-derived suppressor cells (MDSCs). Stromal cells are mainly hepatic stellate cells (HSCs), the primary source of cancer-associated fibroblasts (CAFs). Fibrotic microenvironment in liver is prone to developing into HCC (14). More than 80% of HCC results from extensive liver fibrosis caused by mass CAFs which has been widely reported to be closely related to HCC malignant progression (15). HSCs secrete collagen fibers as well as various components of extracellular matrix (ECM) after being stimulated, strongly contributing to liver fibrosis (16). CAFs-derived soluble factors and exosomes affect cancer cells directly and CAFs can also remodel TME or ECM to regulate HCC progression indirectly (17).

TME is an important window to help us acquaint the mechanism of tumor development and people pay more attention to the studies concerning that the changes of TME promote HCC malignant progression, aiming to discover the most effective therapeutic method to prevent it. Many studies have demonstrated that metabolic alterations about modifying the TME are mainly responsible for the development of resistance to ICIs (18). Intricate cellular crosstalk caused by cellular and non-cellular components in TME contributes to the formation of immunosuppressive TME, promotes tumor cells epithelial-mesenchymal transition (EMT), and increases their resistance to TKIs and ICIs. So cellular crosstalk is a key driver that promotes HCC malignant progression and ultimately leads to poorer clinical prognosis and lower survival rate. Clearly outlining the network of cellular crosstalk in TME will assist in identifying the possible research directions and methods in the future for developing targeted agents with higher efficacy and fewer side effects and designing reasonable schemes of multi-target combination treatments. Based on the above, we reviewed the recent studies about specific mechanisms of HCC malignant progression from the perspective of mutual crosstalk among different types of cells in TME.

## The accumulation and function of dendritic cells

**Figure 2:**
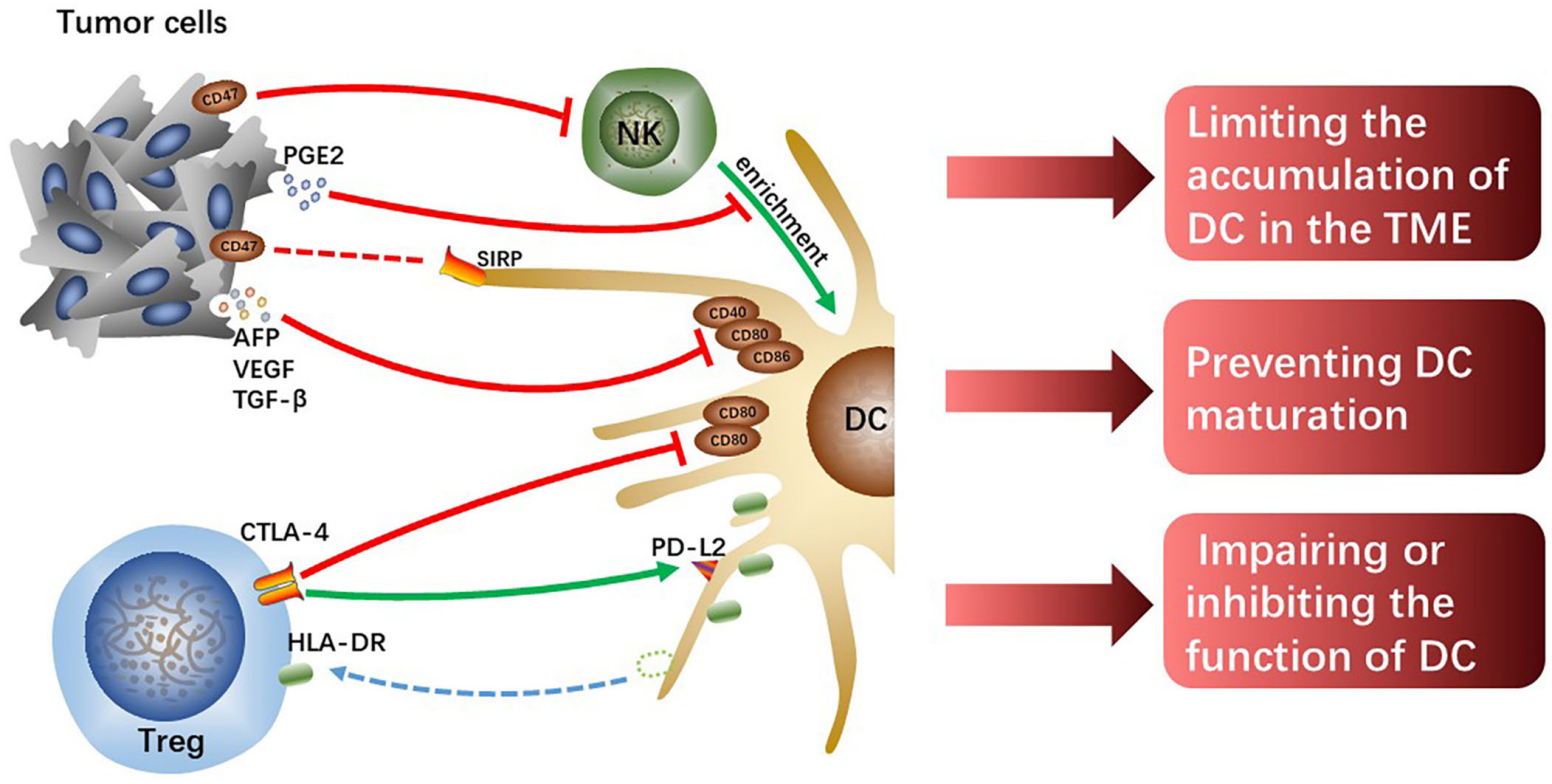
The tumor cells or Tregs interacting with DCs limit the accumulation of DCs, prevent DCs maturation and attenuate their function, which hinders the initiation of effectively anti-tumor immunity.

One of the most important causes of tumor immune evasion is attenuated antigen-presenting ability of antigen-presenting cells (APCs). Dendritic cells (DCs) are the most functional and professional APCs in human body, which can present tumor-associated antigens (TAAs) and activate initial T lymphocytes and then activate the specific antitumor immune responses of the effector T cells (19). Depending on the developmental lineage and differentiation, DC populations exhibit significant variation (20). Conventional DCs (cDCs) play an important role in anti-tumor immunity due to their capacity to present TAAs and release cytokines that modulate T cells survival and effector function. The two types of cDCs—previously known as myeloid DCs—are CD141^+^/CD14^-^type 1 cDCs (cDC1s) and CD1c^+^/CD14^-^type 2 cDCs (cDC2s). The cDC1s are essential for the cross-presentation and activation of CD8^+^ T cells (21). Intratumoral cDC1s recruit T cells, activate and grow tumor-specific CD8^+^ T cells, and enhance T cells effector activity by secreting interleukin (IL)-12 (22–24). The cDC2s are the most common DC type in the human liver, which work by priming T helper (Th) cells to polarize toward Th2 or Th17 and promoting humoral immunity (21, 25). However, the reduction of accumulation and antigen-presenting ability of DCs resulting from crosstalk among cells in HCC TME cannot effectively activate antitumor immune responses, which is one of the important mechanisms causing HCC malignant progression (**Figure 2**).

### DCs inhibition by tumor cells

Chemokine (C-C motif) ligand (CCL) 5 and Chemokine (X-C motif) ligand (XCL)1/2 synthesized and secreted by NK cells are required for early intratumoral cDC1s accumulation and antitumor immunity, however, tumor-derived prostaglandin E2 (PGE2) can disrupt the NK-DC axis (26). In the mouse tumor model, it was found that PGE2 not only inhibited NK cells from secreting chemokines but also induced downregulated expression of chemokine receptors on cDC1, which limited the accumulation of cDC1 in tumor tissues and failed to activate sufficient anti-tumor immune responses, ultimately leading to tumor immune escape (26). However, whether the same mechanism exists in human HCC remains to be further verified, and the specific molecular mechanisms by which tumor-derived PGE2 interacts with NK cells or cDC1s also need to be further explored. In addition, hypoxia induces the production of hypoxia-inducible factor (HIF)-1, a protein that contributes to the heterogeneity of the TME and is linked to the evolution of malignancy in HCC (27). And the expression of the innate immune checkpoint CD47 molecule is regulated by HIF-1α (28). Commonly overexpressed in cancer cells, CD47 is known as a protein that transmits “do not eat me” signals, preventing phagocytosis by DCs and macrophages through the interaction with the signal regulatory protein (SIRP) (29). In addition, Shuai Wang et al. found that CD47 upregulation coincided with reduced CD103^+^ DC and NK cell counts and was linked to a poor prognosis (30). Consistently, the blockage of CD47 increased NK cell activation and recruitment in an orthotopic liver tumor model because of the secretion of chemokine (C-X-C motif) ligand (CXCL) 9 and IL-12 by CD103^+^ DCs and this effect was reversed by CD103^+^ DC depletion (Batf3-/-mice) and IL-12 blocking *in vivo* (30). CD47 may partly explain HCC immune evasion and is a promising therapeutic target.

Moreover, tumor cells-derived IL-10 and IL-6, transforming growth factor (TGF)-β as well as vascular endothelial growth factor (VEGF) prevent DCs maturation, showing a tolerant phenotype with downregulated expression of costimulatory molecules (31). VEGF, TGF-β, and alpha-fetoprotein (AFP) were discovered in the culture supernatant of Hepa1-6-1 expressing higher adhesion molecules, and the culture supernatant significantly suppressed the expression of CD86, CD80, and CD40 on DCs, especially CD86 (32). The cross-presenting capacities and immunomodulatory functions of these tolerogenic DCs with downregulated costimulatory molecules are impaired, failing to effectively activate effector T cells and thus resulting in tumor immune escape. Although the specific molecular mechanisms and signaling pathways that tumor-derived cytokines and growth factors induce downregulated expression of co-stimulatory molecules on DCs are currently unknown, it is undeniable that reversing tolerance DCs to functional DCs is indeed a potential immunotherapy approach.

### Tregs-induced DCs inhibition

Tregs can inhibit immune responses and are always as targets for the treatment of infectious diseases, autoimmune diseases, and cancers (33). Human leukocyte antigen-DR isotype (HLA-DR) expressed on the surface of cDC2 is a key antigen-presenting molecule for activating antitumor effector T cells. However, the level of HLA-DR on cDC2 significantly decreases in hypoxic HCC-TME, which impairs the antigen-presenting capacity of cDC2. Tumor tissue-derived cytokines such as CXCL5 and CCL2 make Tregs and cDC2s enrichment in hypoxic tumor tissue, and it is reported that direct interaction between Treg and cDC2 mediates the loss of HLA-DR on cDC2 (34). Notably, HLA-DR^+^ Tregs increased significantly along with the downregulation of HLA-DR on cDC2 surface and the levels of *HLA-DR* gene expression in both Treg and cDC2 were unchanged, which suggests that Tregs physically extract and ingest HLA-DR from cDC2s under hypoxia. Moreover, it has been demonstrated that these HLA-DR^+^ Tregs exhibit stronger immunosuppressive activity than HLA-DR^-^ Tregs in cervical carcinoma (35). Tregs-mediated downregulation of HLA-DR on cDC2 is a potential immunotherapeutic target for hypoxic HCC, and the antitumor effects of combination with other immunotherapies such as ICIs are expected.

According to studies, Tregs can directly downregulate costimulatory molecules CD80 and CD86 expression or prevent the upregulation of CD80 and CD86 on DC during DC maturation (36, 37), thus weakening the antigen-presenting ability of the DCs. The co-suppressive molecule cytotoxic T lymphocyte-associated antigen-4 (CTLA-4), a transmembrane receptor on T cells, negatively regulates immune responses (38). The effect of CTLA-4 is achieved in part by competing for CD80 and CD86 mainly expressed on APCs with costimulatory molecules CD28 expressed on effector T cells to suppress antitumor immunity. It was previously found that Tregs could downregulate CD80 and CD86 by trans-endocytosis to downregulate their expression on DCs (39). Recent studies have shown that Tregs lacking CTLA-4’s extracellular fraction can also inhibit DCs expressing CD80 and CD86 and that extracellular CTLA-4 function is not crucial for downregulating CD86 and CD80 expression but essential for upregulating the expression of co-inhibitory receptor programmed cell death-ligand 2 (PD-L2) on DCs (40). This novel mechanism of Tregs-mediated DCs inhibition facilitates the discovery of new therapeutic methods to enhance antitumor immunity in HCC.

As intratumoral cDCs are also essential for T cell-based therapies, the low frequency and function of intratumoral cDCs may be partly responsible for the low response rate to ICIs in cancer patients. Hence, increasing the frequency and function of intratumoral DCs is the first step to trigger effective anti-tumor immunity and is probably feasible to combine with other ICIs for HCC treatment. Moreover, it is reported that DCs infiltration might predict the response to camrelizumab and apatinib and tumor recurrence in patients with resectable HCC (41).

## The infiltration and antitumor effects of T cells

**Figure 3:**
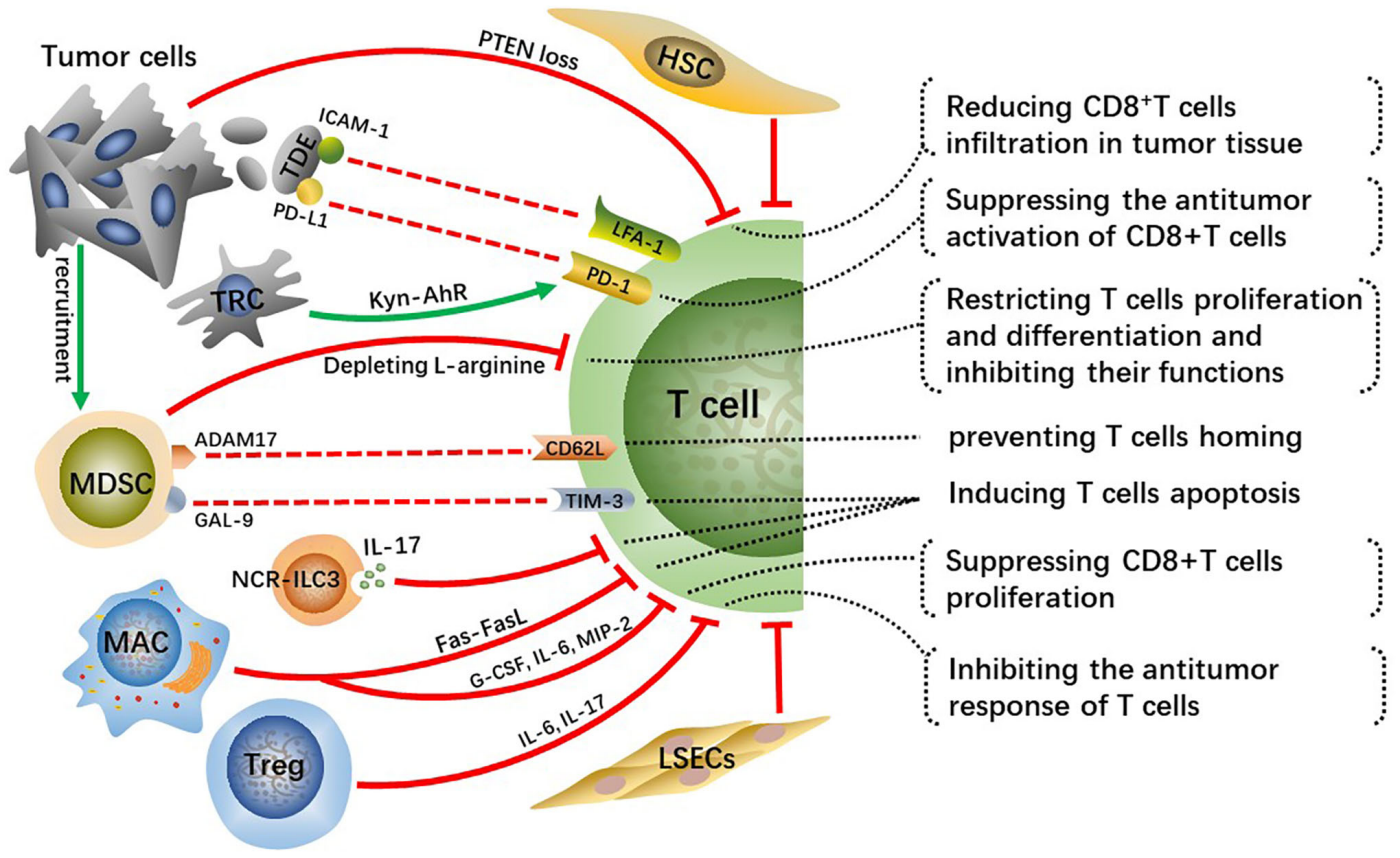
Crosstalk effects of other cells on effector T cells directly or indirectly suppress their infiltration, activation, proliferation and differentiation, which impairs anti-tumor immune effect of T cells and contributes to HCC malignant progression.

CD8^+^ T cells are major lymphocyte subtypes infiltrated in TME and extremely important effector T cells in antitumor immunity. A growing body of research suggested that the downregulation of CD8^+^ T cell activity related to the development of HCC and that patients with HCC may benefit from robust CD8^+^ T cell responses (42, 43). Activated CD8^+^T cells destroy tumor cells by releasing massive granzyme, perforin as well as tumor necrosis factor (TNF). Whereas the crosstalk among tumor cells, immunosuppressive cells, and T cells inhibit the infiltration and antitumor immune effects of effector T cells in tumor tissue, which contributes to tumor cells immune evasion and eventually leads to malignant progression of HCC (**Figure 3**).

### Cancer cells-induced T cells inhibition

Tumor cells occurring specific genetic mutations can reduce infiltration of CD8^+^T cells in TME through certain signaling pathways. For example, phosphatase and tensin homolog on chromosome ten (PTEN) inhibits the activation of PI3K signaling, and downregulation or deletion of PTEN leads to increasing PI3K-AKT pathway activity in multiple cancers, including HCC, thus accelerating tumor malignant progression (44, 45). Studies have found that PTEN loss in cancer cells suppressed the antitumor effect of CD8^+^T cells and reduced T cell transport to tumors in preclinical models of melanoma and was associated with reduced T cell infiltration at tumor tissue in patients (46). Reportedly, PTEN downregulation in the HCC mouse model reduced CD8^+^T cells infiltration in tumor tissue, along with increasing immunosuppressive Foxp3^+^CD4^+^CD25^+^Tregs and upregulating PD-L1 expression on tumor cells. Hence, recovering PTEN expression level in cancer cells can increase the infiltration degree and anti-tumor immune responses of CD8^+^T cells and reverse immunosuppressive TME.

Tumor-derived exosomes(TDEs) with PD-L1 on them inhibit CD8^+^T cells from proliferating and activating (47, 48). Lymphocyte function-related antigen-1 (LFA-1) is one crucial integrin on T cells, whose major ligand is intercellular adhesion molecule-1 (ICAM-1) (49). LFA-1 plays a crucial function in effector T cells destroying tumor cells by binding to related ligands expressed on tumor cells (50). ICAM-1 is present on tumor cell-derived exosomes as well, which can bind to leukocytes, thus preventing them from adhering to activated endothelial cells (51). Interferon (IFN)-γ upregulates ICAM-1 expression on tumor cells and ICAM-1 on TDEs mediates T cell inhibition principally by interacting with activated LFA-1 on CD8^+^ T cells (52). A previous study suggested that PD-L1 is of importance for TDEs-mediated CD8^+^T cells suppression (53). Nevertheless, there is a significantly reduced interaction between T cells and TDEs *via* PD-L1/PD-1 with the absence of ICAM-1, which indicates that the adhesion between tumor-derived extracellular vesicles (TEVs) and T cells mediated by ICAM-1/LFA-1 is a precondition for PD-1/PD-L1-mediated immunosuppression (52). Therefore, targeting TEV-derived ICAM-1 can improve the immune system of cancer patients and has the potential to greatly improve the efficacy of antitumor treatment. This mechanism exists in both melanoma and colon cancer models, while it requires further validation whether a similar mechanism exists in human HCC. Moreover, further analysis of TDEs is essential for understanding their protumor mechanisms and may contribute to developing TDEs-based therapeutic strategies.

In addition, tumor-repopulating cells (TRCs) are reported to promote programmed cell death-1 (PD-1) expression on CD8^+^T cells *via* transcellular kynurenine (Kyn)-aryl hydrocarbon receptor (AhR) signaling (54). Mechanically, TRCs are stimulated by interferon (INF)-γ to produce and secret more Kyn, the latter gets into neighboring CD8^+^T cells and activates AhR, consequently resulting in the upregulation of PD-1. Blockading Kyn-AhR pathway improves the antitumor effectiveness of adoptive T cell therapies.

### MDSCs-mediated T cells inhibition

MDSCs, a group of immature cells with high heterogeneity, originate from bone marrow and synthesize and secrete large amounts of immunosuppressive factors, playing crucial roles in suppressing antitumor immunity (55). Tumor-derived granulocyte-colony stimulating factor (G-CSF), IL-6, VEGF, and CCL2 cause MDSCs migration to HCC-TME (56). Two enzymes inducible nitric oxide synthase (iNOS) and arginase 1 (ARG1) are highly expressed in MDSCs and they cause the depletion of L-arginine, a conditionally essential amino acid related to T cells proliferation and differentiation (57, 58). Mechanically, L-arginine deficiency decreases the levels of CD3 ζ-chain indispensable for the assemble and stabilization of the TCR-CD3 complex on T cells, which weakens the antigen-recognition capability of T cells, as well as TAA-specific immune responses (59). In addition, arginine starvation impairs the formation of immune synapses between T cells and APCs through hindering the dephosphorylation of actin-binding protein cofilin (60). In general, arginine deprivation is one of the main mechanisms for MDSCs promoting HCC malignant progression.

A disintegrin and metalloprotease 17 (ADAM17), a membrane molecule expressed on MDSCs, prevents T cells from homing and being activated *via* interacting with L-selectin (CD62L) on T cells (61). Moreover, MDSCs express galectin(GAL)-9 which can interact with T cell immunoglobulin and mucin domain 3 (TIM-3) and consequently induce T cells apoptosis (62). MDSCs hamper the antitumor immunity of effector T cells through various methods, and controlling the expression of these MDSC-derived factors may greatly improve the antitumor immune responses and effects in HCC patients.

### CD8^+^ T cell repression by other cells

Innate lymphoid cells (ILCs) are a newly discovered family of immune cells that have similar cytokine-secreting profiles as T helper cell subsets and that are critical for host defense against infections and tissue homeostasis. It has been demonstrated that ILC3 lacking the natural cytotoxicity-triggering receptor (NCR^-^ILC3) promoted the development of HCC in response to IL-23 highly expressed in HCC patients and associated with poor clinical outcomes. Furthermore, NCR^-^ILC3 directly induced CD8^+^ T cell apoptosis and limited their proliferation by secreting IL-17 upon IL-23 stimulation (63).

A recent study has revealed that CD11b^+^F4/80^+^ macrophages in the liver metastatic TME are key drivers for inducing CD8^+^T cells apoptosis through Fas-FasL pathway (64). Whether similar mechanisms exist in primary liver cancer such as human HCC needs to be further explored. Reportedly, depleting FasL^+^CD11b^+^F4/80^+^ macrophages by using anti-CSF-1R is prospective but its clinical efficacy for immunomodulatory systemic therapies has not been demonstrated (65). The M2-polarization of macrophages induced by the CCL2 can suppress the proliferation of antitumor CD8^+^ T cells by secreting various cytokines, such as G-CSF, IL-6 and macrophage inflammatory proteins-2 (MIP-2) (66). Prospective studies are desperately needed to identify more reasonable strategies for combinatorial treatment to bypass hepatic resistance and improve the efficacy of systemic immunotherapy.

The research about the cell transplantation model established in immunocompetent mice suggests that HSCs prevent T cell infiltration in tumors (67). Furthermore, activated HSCs reduce responsiveness and cytotoxicity of T cells and increase apoptosis of them *in vivo* (68). Tregs have immunosuppressive activity and play key roles not only in maintaining body immune homeostasis but also in exhaustion of T cells and immune escape of HCC cells. As a kind of anti-inflammatory cells, Tregs can inhibit the response of T cells by producing IL-6, IL-17 and are connected with the poor prognosis of patients with HCC (69). Moreover, increased regulatory DCs induced by CAFs impaired T cell proliferation and promoted Treg expansion *via* indoleamine 2,3-dioxygenase (IDO) (70).

Previous study shows that circulating antigens are captured and cross-presented by LSECs, which contributes to CD8^+^ T cell tolerance rather than immunity (71). Subsequently, it is found that circulating carcinoembryonic antigen (CEA) was preferentially taken up in a mannose receptor-dependent manner and cross-presented by LSECs, but not DCs, to CD8^+^ T cells, which promoted the tolerization of CEA-specific CD8^+^ T cells in the endogenous T cell repertoire through the coinhibitory molecule B7-H1 (72). Moreover, a recent research demonstrates that overexpression of PD-L1 on LSECs inhibits the activation of CD8^+^ T cells and leads to immune evasion of HCC and poor prognosis (73).

In TME, complex cellular crosstalk leads to depletion and exhaustion of effector T cells, which weakens the effect of T cell-based therapies, such as ICI, chimeric antigen receptor T-cell immunotherapy. Hence, blocking various factors leading to T cell depletion and exhaustion is a prerequisite for effective treatment of HCC with other therapeutic methods.

## The roles of CAFs or HSCs in cellular crosstalk

### Crosstalk with HCC cells

HSCs are the main source of CAFs, which are crucial for HCC tumor development, metastasis, and treatment resistance (74, 75). It has been demonstrated that HSCs could be induced to transform to CAFs by HCC cells-derived exosomal miRNA-21 activating PDK1/AKT signaling that directly targets PTEN in HSCs (76). Furthermore, activated CAFs in turn promoted HCC malignant progression by secreting VEGF, matrix metalloproteinases (MMP) 2, MMP9, TGF-β and basic fibroblast growth factor (bFGF) (76). Similarly, exosomal miR-1247-3p derived from high-metastatic HCC cells (HMHs) in the lung metastatic niche reportedly triggered and stimulated β1-integrin/NF-κB signaling pathway in fibroblasts by directly targeting beta 1,4-galactosyltransferase, polypeptide 3 (B4GALT3) and activated CAFs in turn accelerated the development of HCC *via* producing IL-6 and IL-8 (77). A recent study reported that the palmitoylation of hexokinase 1 (HK1) is induced in HSCs after stimulated by TGF-β, thus more HK1 is secreted by forming large extracellular vesicles, which can be absorbed by HCC cells, causing enhanced glycolysis and HCC development (78).

It is found that the upregulation of connective tissue growth factor (CTGF), a matricellular protein secreted by hepatoma cells could activate nearby LX-2 cells (HSC line) and that the activated LX-2 cells promoted HCC cells proliferation by secreting IL-6 that activates STAT3 signaling in HCC cells (79). Similarly, it has been demonstrated that hepatoma cells induced LX-2 cells secreting more growth differentiation factor 15 (GDF15) in an autophagy-dependent manner to enhance hepatoma cells proliferation (80). Blocking the pro-tumor crosstalk between cancer cells and HSCs presents an opportunity for therapeutic intervention against HCC. It is well-known that forkhead box (FOX) proteins play critical roles in amplifying HCC malignancy. CAFs are found to induce FOXQ1 expression and FOXQ1/N-myc downstream-regulated gene 1 (NDRG1) axis is activated in tumor cells, which contributes to HCC initiation (81). Furthermore, the activation of FOXQ1/NDRG1 axis can recruit more HSCs to the TME as a supplement for CAFs *via* inducing pSTAT6/CCL26 signaling (81). The formation of positive feedback loop between CAFs and HCC cells unquestionably accelerates HCC initiation and development.

Nicotinamide N-methyltransferase (NNMT) modulates the metabolism of hepatoma cells and can be induced by activated HSCs. Reportedly, activated HSCs facilitate HCC invasion and migration through upregulating the expression of NNMT that alters the histone H3 methylation on 27 methylation pattern and transcriptionally activating CD44 in tumor cells (82). Although the molecular mechanism of HSCs inducing tumor cells to upregulate NNMT is still unclear, NNMT is a promising prognostic biomarker and therapeutic target for HCC. In addition, tissue inhibitors of metalloproteinases-1 (TIMP-1) secreted by HSCs is upregulated after stimulated by TGF-β, which triggers focal adhesion kinase (FAK) signaling by interacting with CD63 and contributes to proliferation and migration of HCC cells (83).

CD147, a transmembrane protein expressed highly in HCC is a key driver in the metastasis and development of tumor (84). A previous study revealed that CD147 highly expressed on HCC cells mediated the crosstalk between HCC cells and HSCs *via* activating HSCs characterized by high expression of α-smooth muscle actin (α-SMA), collagen I and TIMP-1 as well as increased secretion of MMP2, which in turn accelerated HCC malignant progression (85). *STMN1* known as an oncogene is upregulated in breast cancer, non‐small cell lung cancer, and gastric cancer, which can induce cell differentiation, proliferation as well as invasion and migration in solid tumors (86, 87). Consistently, Rui Zhang et al. also found that STMN1 overexpression in HCC cells could promote cell proliferation, migration, drug resistance, and cell stemness in vitro as well as tumor growth in vivo (88). They also revealed that STMN1 is a bridge mediating complex crosstalk between HCC cells and HSCs by enhancing hepatocyte growth factor (HGF)/MET signal pathway and that STMN1‐induced PDGF secreted by HCC cells may be responsible for activating HSC to acquire CAF features and secrete more HGF (88). Thus, the positive feedback loop for mutual crosstalk between HCC cells and HSCs accelerates HCC malignant progression.

As a pro-inflammatory factor, Follistatin-like 1 (FSTL1) has been reported to promote various cancers malignant progression (89–91). Recent research found that FSTL1 mainly derived from CAFs in human HCC could promote tumor growth, metastasis, and therapy resistance by activating AKT/mTOR/4EBP1/c-myc pathway *via* binding to toll-like receptors 4 (TLR4) on HCC cells (92). It has been reported that c-myc plays a crucial role in hepatocarcinogenesis (93–95) and mTORC1 is vital for the progression of c-myc-driven HCC (96). FSTL1 expression is regulated by TGF-β1 in mouse pulmonary fibroblasts at both transcriptional and translational levels *via* Smad3/c-Jun pathway during fibrogenesis (97). The crosstalk between CAFs and HCC cells *via* TGF-β1 and FSTL1 signaling enhances HCC cells malignancy.

CXCL11 highly expressed by CAFs promoted HCC cells migration, whereas CXCL11 silencing decreased it (98). Concretely, CXCL11 stimulation upregulated circUBAP2 expression in tumor cells, and the later counteracted miR-4756-mediated inhibition on interferon-induced protein with tetratricopeptide repeats (IFIT)1/3 by sponging miR-4756, resulting in upregulation of IFIT1/3 expression that contributed to IL-17 and IL-1β expression, and elevated the migration capability of HCC cells. In addition, CAF-derived cardiotrophin-like cytokine factor 1 (CLCF1) improved HCC cells self-renewal ability through interacting with ciliary neurotrophic factor receptor (CNTFR) enhancing SOX2 signaling and increased CXCL6 and TGF-β expression in HCC cells *via* increasingly activating AKT-ERK1/2-STAT3 pathway (99). Moreover, CXCL6 and TGF-β induced CAFs to produce more CLCF1 *via* activating ERK1/2 signaling, thus forming a positive feedback loop to accelerate HCC malignant evolution (99).

Zhikui Liu et al. demonstrated that stiffness induced HSCs activation *via* CD36-AKT-E2F3 signaling pathway, driving activated HSCs to produce FGF2. Moreover, HSCs-derived FGF2 promoted HCC cells proliferation and metastasis through binding to FGFR1 on HCC cells to stimulate PI3K/AKT and MEK/ERK signaling pathways (100). Reportedly, Sox9/INHBB axis is upregulated in HCC and contributes to HCC development by driving the secretion of activin B to activate the peri-tumoral HSCs through activin B/Smad signaling (101). In addition, Keratin (KRT) 19 is positively associated with the aggressive phenotype of HCC and is upregulated by HSCs-derived HGF through activating c-MET and the MEK-ERK1/2 pathway in HCC cells (102).

The cross-talk between HCC cells and activated HSCs is considered to be important for modulating the biological behavior of tumor cells. We summarized the mediums or means and the corresponding results of the crosstalk between HCC cells and HSCs in **Table 1**. It has been demonstrated that coculturing HCC cells with HSCs under hypoxic conditions enhanced their proliferation, migration, and resistance to bile acid-induced apoptosis compared to coculturing under normoxic conditions (103). How to block the crosstalk between HSCs and tumor and Inhibit cells and inhibit HSC activation deserve more attention in the future.

**Table 1:** The crosstalk between HCC cells and HSCs or CAFs.

Mediums or means	Results	
Exosomal miRNA-21 and miR-1247-3p from HCC cells activate HSCs	Activated HSCs accelerate the development of HCC by secreting VEGF, MMP2, MMP9, TGF-b, bFGF (miRNA-21) and IL-6 and IL-8 (miR-1247-3p)	(76, 77)
More HK1 is secreted by HSCs after stimulated by TGF-β	Enhancing glycolysis and HCC development after absorbed by HCC cells	(78)
HCC cells-derived CTGF activates HSCs	Activated HSCs promote HCC cells proliferation by secreting IL-6	(79)
HCC cells induce HSCs secreting GDF15 in an autophagy-dependent manner	Enhancing hepatoma cells proliferation	(80)
HSCs trigger FOXQ1/NDRG1 axis in HCC cells	Contributing to HCC initiation and recruiting more HSCs into the TME	(81)
HSCs upregulate the expression of NNMT	Facilitating HCC invasion and migration	(82)
TIMP-1/CD63/FAK signaling	Contributing to proliferation and migration of HCC cells	(83)
CD147 activates HSCs	Activated HSCs accelerate HCC malignant progression by secreting TIMP-1 and MMP2	(85)
STMN1‐induced PDGF secreted by HCC cells promotes HSCs to secrete more HGF	Aggravating cancer by triggering the HGF/MET pathway	(88)
FSTL1 mainly derived from CAFs activating AKT/mTOR/4EBP1/c-myc pathway *via* binding to TLR4 on HCC cells	Promoting tumor growth, metastasis, and therapy resistance	(92)
CXCL11 highly expressed by CAFs contributes to IL-17 and IL-1β expression by HCC cells	Elevating the migration capability of HCC cells	(98)
CAF-derived CLCF1 interacting with CNTFR and increasingly activating AKT-ERK1/2-STAT3 pathway	Improving HCC cells self-renewal ability	(99)
HSCs-derived FGF2 stimulates PI3K/AKT and MEK/ERK signaling pathways *via* binding to FGFR1 on HCC cells	Promoting HCC cells proliferation and metastasis	(100)
Sox9/INHBB axis in HCC activates the peri-tumoral HSCs through activin B/Smad signaling	Promoting the metastasis and development of HCC	(101)
HSCs-derived HGF upregulates the expression of KRT19 in HCC	Contributing to aggressive phenotype of HCC	(102)

### Crosstalk with other immunosuppressive cells in TME

HSCs are important for MDSC-induced immunosuppression. A recent study found that activated HSCs could induce monocyte-intrinsic p38 MAPK signaling to enhance reprogramming for the development and immunosuppression of monocytic MDSCs (M-MDSCs) (104). MDSCs overexpressed MMP14 *via* CXCL10/TLR4 signaling. Several studies have demonstrated that overexpressed MMP14 contributed to tumor cells invasion and metastasis (105, 106). It is worth noting that Hui Liu et al. found a novel mechanism of M-MDSC motility that MMP14 regulated by CXCL10/TLR4 mediates M-MDSCs enrichment in liver graft, which promotes HCC recurrence after transplantation (107). Whereas it was found that blocking HSCs-induced intrinsic p38 MAPK signaling in monocytes inhibited the formation of MDSCs and their enrichment in fibrotic liver, which effectively inhibited HCC growth (104). Also, blocking CXCL10/TLR4/MMP14 signaling to inhibit MDSCs mobilization and tumor cells invasion and metastasis will present a great potential for developing novel treatment strategies against HCC malignant progression and recurrence. HSCs also play critical roles in regulating MDSCs migration in HCC. Another research about MDSC migration suggested that HSCs promoted MDSCs migration to HCC TME through SDF-1/CXCR4 axis (108). Hence, targeting activated HSCs in HCC is a potentially beneficial approach for modulating patients’ immune systems.

Endosialin, a transmembrane glycoprotein is demonstrated to mainly express in CAFs in HCC and allows CAFs to recruit macrophages though interacting with CD68 and induce M2 polarization of macrophages *via* regulating expression of GAS6 in CAFs (109). In addition, CAFs could induce macrophages polarize to M2-phenotype TAM (TAM2) and upregulate the expression of plasminogen activator inhibitor-1 (PAI-1) in them by secreting CXCL12, which augmented the malignant characteristic of HCC cells (110).

A lot of previous researches centered on linking various cells in TME with immunotherapy effectiveness. The latest study indicated that TME subtypes of HCC are associated to the immunotherapy efficacy by combining spatial transcriptomics with single-cell RNA sequencing (scRNA-seq) and multiplexed immunofluorescence of anti-PD-1-treated HCC patients (111). It suggested that the tumor immune barrier (TIB) structure consisting of SPP1^+^ macrophages and CAFs near the tumor boundary affected the therapeutic efficacy of ICIs. In addition, it further revealed that the crosstalk between SPP1^+^ macrophages and CAFs contributed to ECM remodeling and TIB formation, which led to reducing immune infiltration in the tumor tissue. Moreover, *in vivo* experiments have verified that SPP1 inhibition in mice with liver cancer resulted in better immunotherapy efficacy with anti-PD-1 (111). Although the molecular mechanism of SPP1-mediated crosstalk between SPP1^+^ macrophages and CAFs is not completely clear and the role of the crosstalk between SPP1^+^ macrophages and CAFs has not been verified in clinical trials, SPP1-blockading is promising for improving the efficacy of HCC treatment with ICIs.

As stroma cells in TME, activated HSCs and CAFs are the core factors that promote the formation of immunosuppressive microenvironment. They directly or indirectly promote the malignant progression of HCC and improve the resistance of tumor cells to immunotherapy. As the roles of HSCs and CAFs in HCC are extensive and complex, continue researches targeting them should not be slack in the future.

## Interactions between tumor cells and TAMs

TAM is a major component of TME playing crucial functions in inflammation-related HCC progression (112–114). TAMs secrete numerous bioactive molecules such as cytokines, growth factors, and MMPs into TME to promote immunosuppression and angiogenesis as well as tumor cells proliferation and metastasis (115, 116). Previously, it is reported that increasing frequency of TAMs correlate with early tumor recurrence in patients with HCC (117, 118) and that macrophage-mediated phagocytosis of tumor cells is inhibited *via* PD-1/PD-L1 (119). A previous clinical trial showed that the combination of tumor-secreted osteopontin (OPN) and peritumoral macrophages is potential to predict tumor recurrence and survival outcomes in HCC patients (120). Recently, Ying Zhu et al. revealed that tumor cell-intrinsic OPN not only facilitated macrophages migrate to TME and polarize to TAMs but also upregulated PD-L1 expression in HCC through activating the colony-stimulating factor-1 (CSF1)-CSF1 receptor (CSF1R) signaling in macrophages (121). Targeting OPN/CSF1/CSF1R axis may be an adjuvant for HCC treatment with ICIs. In addition, It is reported that TAM-derived PGE2 contributes to overexpression of UHRF1, an oncogenic epigenetic regulator, in HCC by repressing UHRF1 mRNA-targeting miR-520d (122). Most notably, UHRF1 upregulates CSF1 expression *via* increasing DNA hypomethylation of the CSF1 promoter, which leads to more TAM accumulation to accelerate HCC malignant progression. Blocking the vicious circle may be an effective approach to the treatment of HCC.

NcRNAs play crucial roles in HCC progression and targeting them may be promising for HCC treatment (123). There are many recent researches about cellular crosstalk between HCC cells and macrophages *via* ncRNAs-dependent manners. For example, TGF-β secreted by M2 macrophages regulates the expression of CD82 in HCC cells *via* upregulating miR-362-3p mediated by binding Smad2/3 to miR-362-3p promoter, which contributes to EMT state of HCC cells (124). In addition, it has been found that TAMs induce the expression of lncRNA H19 and the later increases HCC aggressiveness by stimulating the miR-193b/MAPK1 axis (125). As a kind of crucial medium of cellular signal transmission, the crosstalk between tumor cells and macrophages in exosomes-dependent manners has caused a great upsurge among researchers and here are some of the most recent and meaningful research about them. Exosomal miR-23a-3p released by endoplasmic reticulum-stressed HCC cells increased the level of phosphorylated AKT and the expression of PD-L1 by inhibiting PTEN expression in macrophages (126). Moreover, macrophages stimulated by exosomal miR-23a-3p inhibited T cells function and increased their apoptosis when co-cultured with T cells. The loss-of-function and gain-of-function examinations carried by Xue Li et al. demonstrated that HCC-derived exosomal lncRNA TUC339 is an important component in controlling macrophage activation and M2 polarization (127). In addition, exosomal hsa_circ_0074854 derived from HCC cells can be transferred into macrophages and may contribute to M2 polarization (128). However, the downstream pathways for lncRNA TUC339 hsa_circ_0074854 to function in macrophages and the molecules targeting the pathways should be further explored.

Hypoxia exposure gave rise to high-mobility group box1 (HMGB1) produced by hepatoma cells, which induced TAMs enrichment in TME and upregulation of IL-6, consequently enhancing HCC cells invasiveness and metastasis (129). With persistent hypoxia, HCC cells-derived necrotic debris was reported to induce TAMs to secrete potent IL-1β through the TLR4/TIR domain-containing adapter-inducing interferon-β (TRIF)/NF-κB pathway, which promoted HCC cells EMT and immune evasion (130). Besides, HCC-derived TGF-β increased the expression of TIM-3 on TAMs, which enhanced tumor immune tolerance and stimulated tumor growth *via* NF-κB/IL-6 pathway (131).

Due to a large demand for iron during uncontrolled growth of tumors, iron metabolism is frequently dysregulated in various human malignant solid tumors. HCC cells overexpressed transferrin receptor (TFRC) so that they competed for iron with macrophages and thus limited their iron uptake *via* transferrin (TF)-TFRC axis. Macrophages with low iron tend to polarize to M2-like TAMs by increasing HIF-1α expression (132). Besides, it has been demonstrated that Wnt ligands produced by HCC cells stimulate macrophages polarize to the M2 phenotype by increasingly activating Wnt/β-catenin pathway, which promotes tumor growth, migration and immunosuppression in HCC. Blocking Wnt ligands secretion by tumor cells and(or) Wnt/β-catenin signaling in TAMs contributed to reversing HCC malignant progression (133).

Lulu Liu et al. reported that SPP1 was identified to predict poor survival outcomes in HCC patients by multiomics analysis and that SPP1 was shown to mediate the crosstalk between HCC cells and macrophages based on SPP1-CD44 and SPP1-PTGER4 association by receptor-ligand pair analysis in scRNA-seq (134). Moreover, SPP1 has been demonstrated to promote the polarization of macrophage to TAM2 *in vitro*. Nevertheless, the molecular mechanism that SPP1 mediates the crosstalk between HCC cells and macrophages needs to be further verified *in vivo* experiments and clinical trials.

TAM is one of the important components in TME and plays a key role in the formation of immunosuppressive microenvironment. Tumor cells interact with TAMs, and even form a positive feedback pathway to promote tumor malignant progress. The molecular mechanisms and the corresponding effects of HCC cells interacting with TAMs are listed in **Table 2**. Inhibiting macrophage polarization to the TAM2 is essential to reverse immunosuppressive TME and attenuate HCC malignant progression. How to control the M2 polarization of TAMs and how to block the cytokines and exosomes derived from TAMs are the two central points for future experimental research.

**Table 2:** The interaction between HCC cells and TAMs.

Mediums or means	Results	
PD-1/PD-L1	Limiting macrophage-mediated phagocytosis of tumor cells	(119)
Tumor cell-intrinsic OPN	Facilitating macrophages migrate to TME and polarize to TAMs and upregulating PD-L1 expression in HCC through activating CSF1-CSF1R signaling in macrophages	(121)
TAM-derived PGE2 contributes to overexpression of UHRF1 in HCC by repressing UHRF1 mRNA-targeting miR-520d	Upregulating CSF1 expression and leading to more TAM accumulation to accelerate HCC malignant progression	(122)
TGF-β secreted by M2 macrophages regulates the expression of CD82 in HCC cells	Contributing to EMT state of HCC cells	(124)
TAMs induce the expression of lncRNA H19	Increasing HCC aggressiveness by stimulating the miR-193b/MAPK1 axis	(125)
Exosomal miR-23a-3p released by endoplasmic reticulum-stressed HCC cells	Increasing the level of phosphorylated AKT and the expression of PD-L1 by inhibiting PTEN expression in macrophages and the stimulated macrophages inhibiting T cells function and increasing their apoptosis	(126)
Exosomal lncRNA TUC339	Decreasing macrophage activation and inducing M2 polarization	(127)
Exosomal hsa_circ_0074854 derived from HCC cells	Contributing to M2 polarization after absorbed by macrophages	(128)
Hypoxia-induced HMGB1 produced by hepatoma cells induced TAMs enrichment in TME and upregulation of IL-6	Enhancing HCC cells invasiveness and metastasis	(129)
HCC cells-derived necrotic debris induce TAMs to secrete potent IL-1β through the TRIF/NF-κB pathway	Promoting HCC cells EMT and immune evasion	(130)
HCC-derived TGF-β increased the expression of TIM-3 on TAMs	Enhancing tumor immune tolerance and stimulating tumor growth *via* NF-κB/IL-6 pathway	(131)
HCC cells compete for iron with macrophages *via* TF-TFRC axis.	Macrophages with low iron tend to polarize to M2-like TAMs by increasing HIF-1α expression	(132)
Tumor cells-derived Wnt ligands stimulate M2-like polarization of TAMs *via* Wnt/β-catenin signaling,	Contributing to tumor growth, migration and immunosuppression in HCC	(133)

## Crosstalk among HCC cells and other cells

In human HCC samples from patients with metabolic syndrome, after being stimulated by glucose, insulin, VEGFA or hypoxia, the expression fatty acid binding protein 4 (FABP4), a cytoplasmic fatty acid chaperone protein is upregulated in peritumoral endothelial cells, which promotes hepatoma cells proliferation and migration by upregulating cell cycle-associated pathways and angiogenesis gene expression (135). In addition, there is a research examining the intercellular crosstalk between HepG2 and endothelial progenitor cells (EPCs) in a co-culture system, which revealed that the expression of ephrin-B2, and Delta-like 4 ligand (DLL4) are upregulated in co-cultured EPCs and are associated with increased migration of HCC cells (136). Nevertheless, the molecular mechanisms that HCC cells induce upregulated expression of ephrin-B2 and DLL4 in EPCs and the signaling pathways that ephrin-B2 and DLL4 promote HCC cells migration need further research and exploration. Mesenchymal stem cells (MSCs) have been demonstrated to play critical roles in affecting the aggressive phenotype of several cancers (137–139). A recent study revealed that MSCs could induce upregulation of DNM3OS in HCC cells and accelerate HCC cells proliferation and metastasis through the DNM3OS/KDM6B/TIAM1 axis (140). In addition, liver MSCs-derived S100 calcium-binding protein A4 (S100A4) enhanced HCC cells invasion ability *via* the miR155-SOCS1-MMP9 axis (141).

It is reported that Piwi Like RNA-Mediated Gene Silencing 1 (PIWIL1) was upregulated in HCC and contributed to the proliferation of HCC cells (142). This study not only revealed that HCC cells with upregulated PIWIL1 induced MDSCs transport to the TME and but also demonstrated that HCC cells-derived complement C3 induced by PIWIL1 increased the expression of immunosuppressive cytokine IL-10 in MDSCs by activating p38 MAPK signaling, ultimately leading to HCC malignant progression. The high-frequency of tumor-associated neutrophils (TANs) is correlated with poor prognosis in HCC (143, 144). Neutrophils extracellular traps (NETs) formed by TANs are DNA meshes with associated extracellular cytotoxic enzymes, which partly mediate the crosstalk between cancer cells and TANs (145, 146). A recent study revealed that HCC cells induced NETs formation by secreting cytokine IL-8 and NETs-associated cathepsin G (cG) in turn accelerated HCC metastasis (147). Targeting NETs may be promising to block the crosstalk between tumor cells and TANs and thus prevent HCC malignant development at a certain extent.

Cellular crosstalk among highly malignant HCC cells, low malignant HCC cells and normal hepatocytes also plays important roles in cancer malignancy progression. Oncoproteins are abundant in the exosomes produced by metastatic HCC cells, such as MET protooncogene, S100 family members and the caveolins, which activate PI3K/AKT and MAPK signaling pathways in normal hepatocytes after absorbing them, resulting in upregulation of MMP2 and MMP9 and such enhancing tumor cells migration and invasion (148). Moreover, exosomes produced by HMHs greatly accelerated the invasion and metastasis of low-metastatic HCC cells (LMHs). Reportedly, S100A4 in exosomes produced by HMHs could enhance LMHs’ capability for metastasis *via* activating STAT3 signaling and upregulating OPN expression (149). Alpha-enolase (ENO1) takes part in the Warburg effect by promoting tumor cells absorb glucose and produce lactic acid and is involved in tumor malignant progression and chemotherapeutic resistance (150–152). It has been demonstrated that ENO1 mediates crosstalk between ENO1^high^ and ENO1^low^ HCC cells in an exosome-dependent manner and promotes the proliferation and metastasis of ENO1^low^ HCC cells by upregulating integrin α6β4 expression and activating the FAK/Src-p38MAPK pathway, similarly in ENO1^high^ HCC cells (153). The effects of highly malignant HCC cells on normal hepatocytes and low malignant HCC cells cannot be ignored in the development of HCC.

## Conclusion and expectation

TME is constantly remodeled due to mutual crosstalk among the cells in HCC TME, which is conducive to the maintenance of immunosuppressive microenvironment, and ultimately leads to HCC malignant progression. Although ICIs such as nivolumab, ipilimumab, and atezolizumab as well as TKIs such as sorafenib and lenvatinib have shown impressive efficacy in HCC treatment, especially combination with TKIs and ICIs, only a small proportion of patients responded to them. This may mostly result from tumor heterogeneity and complex cellular crosstalk in TME which leads to constantly remodeling TME and thus developing resistance to therapies.

So for a large number of signal pathways and molecular mechanisms related to HCC malignant progression have been found. With the maturity of scientific theories and the development of biotechnologies, more key targets against to tumor immune evasion and drug resistance will be discovered in the future. At present, most of the researches focus on the mechanical exploration about signal pathways and effective targets of tumor promotion or suppression with single stimulus and single research objective in a single environment background. However, there is an extremely huge and complex network of cellular crosstalk in TME. No matter *in vitro* or *in vivo* experiments, the effects of cell interactions should be taken into account as much as possible.

Hence, researches should attach great importance to co-culture of multiple cells derived from TME and co-stimulation of related multiple factors to better simulate TME and increase the consistency of *in vivo* and *in vitro* experiments. Basic experiments should be closely combined with clinical trials to better serve clinical medicine. Besides individual differences, different carcinogenic inducements and tumor stages may lead to different TME structures with different cell subsets and contents. For example, it has been revealed that a metabolic network-driven approach can stratify the HCC tumors into three distinct tumor subtypes (154). The latest study has revealed that TME subtypes of HCC are associated with the immunotherapy efficacy (111). Therefore, researching the structures of TME subtypes in categories and exploring the molecular mechanisms of cellular crosstalk based on that are expected in the future for the development of precision medicine.

## Author contributions

PL and LK were responsible for study concept and design. PL, LK, and YL were responsible for study selection and material collection. XL oversaw the project and revised the important intellectual content of this manuscript. PL, GL, and JX drafted the manuscript and contributed to drawing the mechanism diagrams. All authors participated in the interpretation of the results and preparation of the manuscript and agreed to its published version. All authors contributed to the article and approved the submitted version.
